# Use of broad consent and related procedures in genomics research: Perspectives from research participants in the Genetics of Rheumatic Heart Disease (RHDGen) study in a University Teaching Hospital in Zambia

**DOI:** 10.1080/11287462.2019.1592868

**Published:** 2019-03-24

**Authors:** Oliver Mweemba, John Musuku, Bongani M. Mayosi, Michael Parker, Rwamahe Rutakumwa, Janet Seeley, Paulina Tindana, Jantina De Vries

**Affiliations:** a Department of Health Promotion and Education, University of Zambia, Lusaka, Zambia; b Children Hospital, University Teaching Hospitals, Lusaka, Zambia; c Department of Medicine, University of Cape Town and Groote Schuur Hospital, Cape Town, South Africa; d Wellcome Centre for Ethics and Humanities (Ethox), Nuffield Department of Population Health, University of Oxford, Oxford, UK; e Medical Research Council/Uganda Virus Research Institute and London School of Hygiene and Tropical Medicine Uganda Research Unit, Entebbe, Uganda; f Department of Global Health and Development, London School of Hygiene and Tropical Medicine, London, UK; g Navrongo Health Research Centre, Ghana Health Service, Navrongo, Ghana; h Department of Medicine, University of Cape Town, Cape Town, South Africa

**Keywords:** H3Africa, broad consent, genomics, bio-banking, Zambia

## Abstract

The use of broad consent for genomics research raises important ethical questions for the conduct of genomics research, including relating to its acceptability to research participants and comprehension of difficult scientific concepts. To explore these and other challenges, we conducted a study using qualitative methods with participants enrolled in an H3Africa Rheumatic Heart Disease genomics study (the RHDGen network) in Zambia to explore their views on broad consent, sample and data sharing and secondary use. In-depth interviews were conducted with RHDGen participants (*n* = 18), study staff (*n* = 5) and with individuals who refused to participate (*n* = 3). In general, broad consent was seen to be reasonable if reasons for storing the samples for future research use were disclosed. Some felt that broad consent should be restricted by specifying planned future studies and that secondary research should ideally relate to original disease for which samples were collected. A few participants felt that broad consent would delay the return of research results to participants. This study echoes findings in other similar studies in other parts of the continent that suggested that broad consent could be an acceptable consent model in Africa if careful thought is given to restrictions on re-use.

## Background

Genomics is now a well-established approach to strengthening health research capacity in Africa, with researchers across the continent engaging in exploring genetic factors that play a role in disease causation including how to prevent, diagnose and manage diseases (Peprah, Xu, Tekola-Ayele, & Royal, 2015; Wonkam & Mayosi, 2014). Such research has been conducted in conjunction with an evolving understanding of the ethical challenges that should be considered in its execution (De Vries et al., 2011; Wright, Koornhof, Adeyemo, & Tiffin, 2013). As is the case for health research more broadly, the ethical topic that has received the most attention is consent, and an increasing number of scholars have identified challenges and opportunities for ensuring valid, informed consent for African genomics research (Tindana & De Vries, 2016). Such literature has described the difficulty of adequately explaining complex genomics research methods (Marshall et al., 2014; Tekola et al., 2009), the risk of diagnostic misconception particularly when recruiting healthy population controls (Masiye, Mayosi, & de Vries, 2017) and the design of consent forms (Munung et al., 2016). Many of the challenges relating to obtaining valid consent for genomics research are rooted directly or indirectly in poverty, including for instance participants’ limited access to quality healthcare and low health and research literacy. But whilst these are features of many participants enrolled in African genomics research conducted in more rural areas, some evidence from urban Nigeria cautions against making generalised assumptions about prospective participants’ research literacy (Marshall et al., 2014).

One limitation of the African literature around informed consent for genomics research is that it reports on findings from only a limited number of countries, with few papers reporting on the experiences and views of research participants based in low income countries in southern Africa. Specifically, there is no evidence originating from Zambia; a country which adopted a particularly stringent approach to consent for genomic research (Chanda-Kapata, Kapata, Moraes, Chongwe, & Munthali, 2015).

One important feature of much genomics research is its reliance on large numbers of samples to detect small genetic effects. Once collected and stored, there is the possibility for such samples and data to be reused for valuable research that may or may not be related to the condition for which they were originally collected. Together, these features mean that genomics research is often collaborative (De Vries et al., 2011). If samples and data are to be legitimately available for future research, it is essential that the consent process at the time of collection allows for this possibility. Where this includes the potential for research that is valuable but not foreseeable at the time of consent, this suggests the need for the initial consent to be “broad”.

Broad consent is consent for future studies but with certain restrictions on the nature of future studies that may be conducted and on the decision processes for allowing future studies to take place (Grady et al., 2015; Tindana & De Vries, 2016). The use of broad consent for genomics research raises a number of important ethical questions, including for instance whether such consent should be seen as ethical considering that even though future uses are constrained to some extent, people are not given full and specific information about potential studies (Hofmann, 2009). A relatively recent shift in ethics debates surrounding genomics research has seen greater acceptance of broad consent as the “best compromise” consent model to be used when recruiting participants (Grady et al., 2015; Jao et al., 2015; Tindana & De Vries, 2016), possibly because it can be considered “consent for governance” (Sheehan, 2011). Yet there are pertinent questions around the use of broad consent for the recruitment of participants for genomics research in Africa, relating for instance to the acceptability of such a consent model for research participants and comprehension of difficult scientific concepts.

In order to explore these and other issues, we conducted empirical research with a range of stakeholders to explore their views on the consent process for genomics research broadly. Our project forms part of a multi-site qualitative study in Ghana, Uganda and Zambia. In this paper, we report on the results of a study conducted in Zambia where we conducted interviews with participants (or family members for paediatric patients) in a genomic study focusing on rheumatic heart disease (RHD). The Genetics of Rheumatic Heart Disease (RHDGen) study was conducted under the umbrella of the H3Africa Consortium (H3Africa Consortium, 2014) and involved research partners in 8 African countries including Zambia. It used a broad consent model seeking consent for the primary study and for sharing and future use of data. Specifically, the consent forms stated that “It is now common that genetic information is shared with researchers around the world. The benefit is that many researchers can use the same information for different research projects. We would also like to share your genetic information and some of the clinical information with other researchers for other projects after we finish our study. If you participate in this study, you will also need to agree to share your genetic information for other research in the future.”

RHD is caused by an aberrant immune response to *Streptococcus pyogenes* throat infection, and affects mostly children and young adults (Karthikeyan et al., 2012). The condition is strongly poverty-related (Carapetis & Zuhlke, 2011) – whilst virtually eradicated in wealthy countries and communities, it continues to be a devastating illness in poor communities in Africa, often resulting in premature death (Zühlke et al., 2016). It is estimated that RHD could account for up to 1.4 million deaths per year and is one of the leading non-communicable diseases among children and young adults in low- and middle-income countries (Carapetis, Steer, Mulholland, & Weber, 2005; Watkins et al., 2017). Whilst surgical operations are available to replace damaged heart valves, in Africa such operations are currently only routinely available to public sector patients in a few countries such as Namibia, Senegal, and South Africa. Zambian patients do not normally have access to such operations. Genetic risk factors play a considerable role in the aetiology of RHD (Engel, Stander, Vogel, Adeyemo, & Mayosi, 2011), providing a strong rationale for conducting large-scale population based genomic studies on this condition.

## Study methods

### Study site

The study was conducted at the Department of Paediatrics at the University Teaching Hospital (UTH) where the Zambian RHDGen study was based. This is a tertiary and referral hospital for specialised care, based in Lusaka, which is the Zambian capital. Most of the participants therefore live in urban and peri-urban areas.

### Study sample

In this paper, we report on findings resulting from in-depth interviews with (or family members of paediatric patients) individuals enrolled in the RHDGen study (*n* = 18), with study staff involved in enrolment (*n* = 5) and with individuals who refused to participate (*n* = 3). We enrolled adult RHD patients, family members of paediatric RHD patients, and healthy population controls approximately one year after they consented to participate in the RHDGen study. Table 1 gives an overview of these participants. We identified patients using a register of participants in the RHD study. We contacted patients by telephone. In addition to the interviews reported in this paper, we also conducted 20 interviews with regulators, researchers and ethics committee members, but we report on the results from that component of our research elsewhere. Due to delays in obtaining ethics approval for this study, we started recruitment for this study when the RHDGen study had already completed enrolment. This means that we spoke with our interviewees sometime after they had agreed or refused to participate. The timespan between the date of RHDGen enrolment and the date of the interview ranged anywhere from 10 months to 20 months. When enrolling people at this study, we explained the concept of broad consent to interviewees when administering informed consent and during the interviews. Our explanation included the difference between broad consent and blanket consent by emphasising that blanket consent does not restrict the use of future use of samples while broad consent does. Broad consent was also distinguished from specific consent by indicating that the latter is concerned with the particular study for which samples and data are collected. Interviews with RHDGen participants and non-participants were conducted in Nyanja (*n* = 15), Bemba (*n* = 3), Tonga (*n* = 1) and English (*n* = 2) and translated to English. All interviews with staff were conducted in English.

**Table 1.** Number and type of interviewees recruited.

Type of interviewee	Enrolled in RHDGen?	Number
Study staff	No	5
Family member of paediatric RHD patient	No, but consented to enrol their child	4 (2M, 1F plus an interview with both parents present)
Adult RHD Patient	Yes	4 total (4F)
Healthy control, unrelated to RHD patients	Yes	4 total (3M, 1F)
Healthy control, related to RHD patient	Yes	1 total (1F)
Individuals who refused to participate	No	3 total (1M, 2F)

Note: F = female; M = male.

### Data analysis

All interviews were audio-recorded and transcribed verbatim. Where interviews were not conducted in English, they were translated simultaneously by two research assistants and we verified the translation independently. Following transcription, text files were imported into NVivo 11 (QSR International Pty Ltd, 2015). This involved initial open coding of 5 transcripts by two researchers (OM and JdV). Following discussion, we developed a hierarchical coding scheme together with a codebook describing each code and its relationship to overarching study themes. Interviews were then coded by the researchers and two research assistants. Two researchers (OM and JDV) together analysed and interpreted coded data using the Framework Method functionality embedded in NVivo 11 (Gale, Heath, Cameron, Rashid, & Redwood, 2013; Parkinson, Eatough, Holmes, Stapley, & Midgley, 2016; Smith & Firth, 2011). We discussed data interpretations with the wider study team.

## Findings

This study set out to explore Zambian RHDGen participants’ views on one model for consenting used in genomics research called broad consent. A number of inter-related themes on broad consent emerged from the interviews. These include views on broad consent; sample and data sharing and bio-banking; consent for research on the stored specimen for which broad consent was not obtained; concerns about blood collection and use; and motivation to participate in the RHDGen study and feedback of findings. Table 2 summarises the key findings on each of these themes.

**Table 2.** Emerging themes.

Theme	Key findings
Views on broad consent	Positive view of broad consent if the participant sufficiently informed General feeling was that broad consent should be restricted only to future studies that help understand the outcomes of the original study and the health condition of participants Some people were sceptical about broad consent because it meant that the return of results would take long
Sample and data sharing, and bio-banking.	Sample and data sharing supported if it ultimately benefits the patients and community at large Sample and data sharing supported if it increased the power, validity and reliability of the study Sample and data sharing supported if the samples are anonymised
Consent for research on stored specimens for which broad consent was not obtained	Researchers should inform and get consent from participants who provided the samples If impossible to contact participants, regulatory institutions can review proposed studies and approve Research can go ahead if it benefits the society and individuals who gave the sample Participants have no control on samples once given
Concerns about blood collection and use	Fear that blood will be used for satanic rituals when shared with unknown (foreign) researchers Not sure how the blood will be used because they have no control once the blood is given Fear that blood will be sold for profit Worry about loss of blood
Motivation to participate in the RHDGen study and Feedback of findings	Participation will help understand the causes RHD condition and find solutions to the management and treatment of the condition access to specialist treatment and surgery Participation provided an opportunity for free screening and diagnosis for heart conditions Participation created a network for sharing experiences among RHD patients and care givers Participants keen to know the results including individual genetic test results and other incidental findings relating to participants’ health Feedback on general results will maintain trust and promote future recruitment of study participants Feedback of results could explain the RHD condition and how to manage it in future

### Views on broad consent

In general, most participants seemed to have a positive view of broad consent on condition that information on the reasons for storing the samples for future research use was disclosed. Most participants were able to relate broad consent to what they remembered about the RHDGen study. The participants including one of those who opted not to participate in the RHDGen study, remembered quite a lot of detail about the RHDGen study including that RHD affected the valves of the heart, and that the RHDGen study aimed to establish the genetic causes of RHD which involved the exporting and keeping of samples for a long time. They also indicated that they were told that feedback of study results would take long.

Several participants also argued that if the research participant is informed from the onset when the sample is being collected that researchers plan to use the specimen for other research in future, they would not have a problem with that. One participant argued:

[broad consent] is acceptable when that person is counselled; then that person can be convinced and accept but if they refuse that’s up to them as an individual but first, start with counselling, you are counselled you are told so that you know what you have gone there for  …  it is up to me to accept or refuse. (IDI 11 – Female unrelated control)

Importantly, some participants felt that broad consent should be restricted in a way such that the planned future research should be spelled out and should not deviate so much from the original research the participant consented to. One participant argued that further research should only be permitted if it helps to understand the original health condition of the patient who gave the sample.

When you are talking about broad consent and if declaration is made at that point then if I have allowed you to go ahead then you may go ahead with that … but I still feel that the concentration really should be more of what affects the person that you got the sample from, should be the main focus … I think it is good to stick to what the [original] research is all about but if the other researches are helping to understand more, then there is nothing wrong with that. (IDI 5 – Adult female patient)

Yet other participants were uncomfortable with broad consent because they felt it would delay the return of research results to participants – which would indicate some sense of therapeutic misconception.

Broad consent is not a good idea, when they collect blood, they need to give us results there and then so that if there is need to offer help, they do it fast. There are a lot of diseases found in the blood like hepatitis, diabetes, heart disease and many others. But if that blood is going to [other countries], they would not help us fast enough. In fact, what we want us people with heart problems is that there should be quick treatment. (IDI 8 – Adult female patient)

The therapeutic misconception was not only limited to those who chose to participate in the RHDGen study. One participant who opted not to participate in the RHDGen study gave as the main reason for not participating, that their son was getting better; and hence that there was no need to participate in the research. Here is an exchange between the interviewer and one of the respondents who declined to participate in the RHDGen study.

Interviewer: I want to know, how much understanding did you have about the study?

Respondent: I think I understood it well and we wanted to participate, I was with my husband, he also agreed but because our son was getting better so he [husband] did not see the need to participate in research, because he [our son] was improving.

### Sample and data sharing, and bio-banking

As indicated above, the notion of broad consent has primarily been developed in relation to the requirement for the storage and sharing of samples and data in collaborative genomics research. In principle, most of our interviewees supported the sharing of samples and data among researchers. There were some variations and similarities on the reasons advanced for supporting sample sharing and data sharing. For example, some participants supported both sample and data sharing because they thought this would improve the likelihood that researchers could learn something that benefits the patients and the community at large.

… we want to know where the disease is coming from. So if they get the sample and have shared, it is just okay because we are want to find out what is bringing the diseases  …  that is what is important to discover the diseases to help other people and the person you got the sample from. (IDI 4 – Male parent)

One highly educated participant (with tertiary education) also argued that sharing samples and data increases the sample size for research which increases the power of the study conducted from such samples and data, hence improving the reliability and validity of the study and producing results that will benefit patients and generally health care.

You see when you are doing research, I always believe in a wider range of samples to try and get the true picture … So, to narrow the percentage error, it is good to share the samples to come up with realistic results because I believe that you are doing this to try and find solutions to many people that are suffering out there, and come up with the best medications. (IDI 5 – Adult female patient)

There were also some participants who did not mind their samples and data being shared as long as this was done in an anonymised form.

Like you have said you don’t put names; you don’t put anything for you to identify who the blood belongs to; it is ok because other scientist will not know that it’s us. (IDI 15 – Female parent related control)

Others did not have problems with sample sharing because they related it to giving blood donations, which was in their mind routine and “normal” as a way of saving other people’s lives.

On bio-banking, most participants felt that it was a good idea because it would provide an opportunity to use the samples to conduct research that will find solutions to medical problems and diseases.

It is not a bad thing because you find that when you look at this critically, even when there is a new disease, for them to find medicine, some people would have died, sometimes they could have gotten samples from those people, and they go do research to find a cure for that disease. So on that issue where they get blood samples and they keep them for three to four months it is not a bad thing because even those who come to learn from the samples, they are the ones who are helping us. (IDI 13)

Some participants also felt that biobanks would provide an opportunity or resource for training “future” scientists and students. Two participants put it this way:

Biobanks, I think it is a right idea, specimens are stored in a safe and in good condition. Many students coming by will learn, do research and definitely they will find an answer, one day they definitely find an answer. It is a very good idea. (IDI 12)

On banking that blood you got for others to investigate and learn from it; I see it as a good idea because our children will learn from that blood you are storing in there, there are children who are still going to school, those children coming in the next generation, you will teach them using the same blood rather than throwing it away … if you store it, it’s good it will help us. (IDI 15)

### Consent for research on stored specimens for which broad consent was not obtained

The participants also discussed ways to approve or monitor future research on existing archived specimens originally collected from participants for a different purpose with specific consent. Most participants argued that they would prefer that the researcher informs and re-consents the participants who provided the sample. However, some participants argued that regulatory institutions such as ethics committees and holders of specimens such as researchers should review and approve such research especially when it is difficult to trace the participants who gave the specimen.

[If] am not here and I have given you my samples, I stay far, and you are in a different place  …  I think it just okay for you just get the sample and learn from them and maybe from the place where that sample is kept you can get the permission from them because on my part you already got the samples from me. (IDI 4 – Male parent)

There was one participant who thought that research should be allowed to go ahead even without consent as along as it benefits society and the individual concerned stand to benefit from such research.

They can just learn from them [samples] because still there are kids that will be getting sick. It is not just our [daughter], the other children that can get sick, many people are sick and a lot are still coming because this is the condition [RHD]. (IDI 2 – R2 – Female parent)

Another participant felt that they had no control on the specimen that is already given because it is already in the hands of the researcher.

Since they have them already, even if we say that we deny, they may use them. There is no way we can object because they have the samples already. (IDI 2 – R1 – male parent)

### Concerns about blood collection and use

Blood is a common specimen used and shared in genomics research. Many of the participants in this study expressed concerns about the blood collected for the RHDGen study, which relates directly to their views on sample storage and sharing. The general concern was that blood may be abused for satanic rituals especially when given or shared with researchers who are not “known” (not Zambian) to participants. If shared with unknown researchers, some participants argued that they would not know how the blood samples would be used and would have no control over what can be done. The absence of trust and control leaves room for abuse.

Other people they are not comfortable. You know why, because people are scared in this world we are living in, blood samples are used for so many different aspects, not only medical purposes. [so] other people it was not easy to just give blood. The researchers had a tough time to convince them because people don’t just give blood anyhow because it can be used for wrong purposes. (IDI 12 – male unrelated control)

These concerns about blood were also used by potential participants to discourage others from participation.

We were discouraged to say when you give too much blood you faint, others said that your blood will be taken into Satanism. So for me I said “no, even if it’s Satanism for us we want to give blood to help people”. (IDI 14 – male unrelated control)

The participants argued that this view was not limited to research participants but was common in the community. This includes some churches, who participants described forbids the giving of blood for fear of it being used for satanic rituals. There are also some participants who were concerned that researchers may be selling the blood for profit while one participant was worried about fainting due to loss of blood.

Most of these concerns about blood were echoed by staff involved in the recruitment of participants in the RHDGen study. They indicated that concerns on how the blood will be used were the main reason potential participants declined to participate in the study. The staff indicated that both those who refused to join the study and those who joined were uncomfortable that their blood was to be shipped to other countries, which may explain why so many participants remembered this particular aspect of the RHDGen study.

Yet even amidst rumours of Satanism, because of the severity of the condition, one parent expressed determination to participate in the study, hoping to find a solution to the condition of their daughter.

These are the things people are saying for sure [discouraging to donate blood for fear of Satanism] but for us we want our child to get better even if they discourage us we are just looking for solution our child … So I said the Satanism didn’t matter to me, what mattered was to find the cause of the problem [RHD]; if it’s with us the parents [genetic] or if the problem came on its own. (IDI 15 – Female parent related control)

### Motivation to participate in the RHDGen study and feedback of findings

All participants were aware that study participation was voluntary and many participants indicated that they participated in the study because of the potential benefits that may come with the study. Motivations for participating in the study varied between those who were patients or parents of children with RHD and healthy participants. Those who were (parents of) patients hoped that through the study they would facilitate access to specialist and advanced treatment for their condition. Those who participated as healthy controls saw participation as an opportunity to know how healthy their heart was because the study involved a detailed examination of the heart and free medical consultation with heart specialists if any abnormalities were found.

Yet against this background of expecting a personal benefit, many participants also described altruistic reasons for participating, often linking these to descriptions about the devastating nature of RHD for children and expressing the hope that participation would help researchers learn more about the causes of this illness as well as developing new treatments for future generations.

One unexpected influence of the RHDGen study is that it led to the creation of a support network of patients where they are able to share experiences about the RHD condition and best ways to manage and cope with the illness.

We talk about these things when we meet, it’s like we become one family and we know each other. So we are happy that the research you are doing, you should continue investigating to find out the cause of the problem and then a solution can be found … (IDI 15 – Female parent related control)

This social network extended to the staff on the RHDGen study. The lead staff member reported how the relationship with the participants especially RHD patients and their parents evolved over the course of the study to a level where they continued consulting each other and seeking comfort. She indicated that patients have remained “attached” to her and other research staff and continue to hope that the RHDGen study would provide solutions to their plight.

Because of the shared experiences with the RHDGen study and expected health benefits that motivated participants to join the RHD study, almost all participants were keen to know the study results and thought that feedback should include results of the genetic tests for individuals who participated in the study. Here is how one participant put it.

Of course these samples were going far; it’s not near and a number of test were going to be done to try and establish, so when I heard that, I was interested to say I might get some information on how I found myself like this [with RHD] … Actually, am looking forward to hear what the outcome is. And if there any recommendations on how I should live after that? [The research staff] mentioned that in future they will get in touch with me but it has been quiet. (IDI 05 – Adult female patient)

Whilst all participants describe their expectation that they would receive some results, only three spoke about receiving general study feedback (in conjunction with individual-level results) and the others only spoke about receiving individual research results. With regards to receiving general study findings, participants articulated the view that feeding back such findings would be a matter of courtesy and would play a role in maintaining trust, which could promote future recruitment of study participants.

For us guys who volunteered, its best that you get back to us and tell us more because we are interested, that is why we volunteered, so it is best that you come back to us, even sensitise us more so that we can feel we are part of this research. (IDI 12 Male Unrelated Control)

With regards to the return of individual research results, participants expect results that pertain directly to RHD, as well as information pertaining to other conditions found in their blood. When probed, this includes information about other conditions such as diabetes and HIV.

Respondent: When the results come out, I would want to be told what have been found in the child [patient].

Interviewer: So if you gave the sample for testing for the heart, and they test for example diabetes would you want to know?

Respondent: Yes, I would want to know (IDI 03 Female Parent)

Participants described an obligation on the part of researchers to inform them of any pertinent results identified in the project if these are relevant to their health.

I think that since you are the only one who knows that this blood belongs to Agnes’ mum that has such a problem, you are supposed to call me and find a way to prevent it. (IDI 15 Female Related Control. “Agnes” is a pseudonym)

One challenge is that the recruiters used the family inheritance analogy to explain the genetic component of our study – and as we showed above, many participants remembered this component of the RHDGen study. But this explanation may also have confused participants, in that they understood that the study was aiming to understand why they or their children developed RHD.

I did understand what they said, to know where the problem for Alex is coming from. (IDI 02 Parents of paediatric RHD patient themselves enrolled as related controls. Alex is a pseudonym)

People expect feedback for a number of reasons, including an expectation that it would reveal information about why their condition developed and how it could be managed in the future.

Yes, the results delay to come back and they don’t let us no know the results from the blood samples they got and what the way forward will be. (IDI 08 Adult female patient)

The staff who recruited the participants for the RHDGen study confirmed participants’ desire to know their results. They argued that participants, particularly those with RHD or parents of children with RHD were desperate for solutions to their condition and hence needed immediate feedback on their results. The hope from the participants is that knowing the results may help find solutions to their condition. This was despite explaining to the participants that they should not expect individual benefit and that the study would take long to complete. The lead nurse on the team added that she continued receiving calls from participants wanting to know their results, one year after the study closed.

Sadly, by the time we conducted the interviews, three of the paediatric patients who had enrolled in RHDGen had passed away, illustrating how deadly this disease is in the absence of heart valve replacement operations. The family members of those children expressed a desire to receive results that may help them understand more about the condition in general, and about whether anything could have been done to prevent the death of their child. Understandably, they also expressed frustration with the researchers and their institutions that the results they were expecting were not given “more quickly”.

That’s what kept coming to my mind, but I never just wanted to show it. I was just trying to calm down to say just wait, the results will come out but all in all, what we wanted is the attention to the boy; it was supposed to come because those researches whatever you were doing, to me, it was taking time for those results to come out and to be communicated to the involved people. (IDI 01 Family member of deceased RHD patient)

I have noticed that from the time they collected blood samples, it could have been good if the results were out and that main problem known before she even died, maybe something could have been done. (IDI 07 Female Parent to deceased RHD patient)

Whilst in international debates around the return of individual genetic research results emphasis has been placed on autonomy as a guiding principle to determine whether results should be fed back, our participants all seemed to be of the opinion that individual results pertinent to the health of the participant ought to be fed back. The reason for this, seemingly, is an expectation of reciprocity – that it is part of the researchers’ duty to care broadly for the health of research participants, and to “help” them if they can. One respondent who refused to participate in the RHDGen study emphasised that participants in genomics research should benefit from it as much as the researchers do.

Let’s say you are studying the RHD, you find the cure for RHD, there will be money involved in that, so those people who will be making those medicine will be selling to the entire world and make a lot of profit. The person who contributed blood is not getting anything and is not even remembered … Researchers must not forget the contributor of the blood because they are taking part of their life, they are giving you [researchers], for someone else to benefit.

## Discussion

In general, participants in this study found the use of broad consent reasonable if it is clearly explained to them at the onset of the study. By that, participants seemed to mean that the intention to store and share samples and data for future studies should be clearly explained to and understood by participants before signing the consent form. These findings are consistent with other studies. For example, in a study based on a review of the literature on broad consent in the context of genomics and bio-banking research, Tindana and De Vries (2016) found broad consent and sharing of samples and data to be acceptable in different contexts in low- and middle-income countries.

However, our study went further to reveal that acceptability of broad consent may be contextual to participants’ health problems and specific needs. For example, our study shows that one frequently given reason for supporting broad consent and sample and data sharing was the hope that this would accelerate knowledge generation and the development of new treatments for Rheumatic Heart Disease, for themselves and others. Such expectations were linked to the reported gravity of the condition – in our group of 11 RHD patients, 3 children who had enrolled in the RHDGen study a year previously had since passed away – and the very costly treatment options available to participants. In the light of such a lethal and largely untreatable condition in the Zambian context, participants expressed support for anything and everything that could help alleviate their plight, and that of others.

As indicated earlier, broad consent is not open-ended or “blanket” consent, but rather consent for future use *with restrictions* (Grady et al., 2015; Tindana & De Vries, 2016). In the literature, it is not currently clear what level of restrictions would be acceptable for African research participants. Arising out of concerns of sample misuse as well as considerations of fairness, most participants suggested that future studies should be limited to the condition for which samples were collected. They also suggested that there should be concrete ways to ensure that any benefits from future studies would trickle back to participants, other patients with RHD, or Zambia. This was true for generic study findings and possible treatments, but also for individual study results including individual genetic findings. This resonates with other studies conducted on the continent. For instance, van Schalkwyk, de Vries, and Moodley (2012) also found that participants supported re-use that was beneficial to others in their immediate environment. Jao et al. also found that participants in Kenya considered broad consent as the “best compromise” to balance scientific utility with ethical principles, but with the requirement that future studies are of benefit to their communities.

On some level, participants seemed to talk about the feedback of individual research results as a proxy to reciprocity – or, as part of the expectation that researchers will be cognisant of and seek to alleviate their poverty and medical needs insofar as possible. Some of the participants we recruited are too poor to afford even the most basic forms of medication or medical consultation. They do not have access to heart operations that would make their conditions manageable. Against this background, participants emphasised the importance that researchers think carefully about how their project can accommodate patient needs, for instance through feeding back pertinent information that would help them access better treatment or plan their lives. One important implication is that African genomics research projects need to develop a clear protocol and counselling strategy for participants including a plan for disclosure where results have implications for the family or community.

It was also clear from our study that participants struggled to separate the research healthcare components of the RHDGen study, possibly because the study’s primary investigator was also the treating cardiologist for many participants. For example, with regards to the return of research results, we found strong evidence of diagnostic and therapeutic misconception, which is the belief that blood samples collected for genomic research are actually taken for diagnostic or treatment purposes (Masiye et al., 2017). Whilst Masiye et al. describe diagnostic misconception in healthy participants enrolled as controls, in this study we find this misconception to equally exist with RHD patients and their family members.

The findings from this study add to the body of knowledge for developing evidence-based legal and regulatory frameworks in genomics research in Africa. For example, in Zambia, the 2013 Health Research Act (HRA) prohibits the use of broad consent by prescribing that consent needs to be specific and may not be obtained for unspecified future studies (Chanda-Kapata et al., 2015; de Vries et al., 2017). Whilst the HRA was partly developed to protect participants from exploitation, this study raises a debate on whether the assumptions that informed the development of the HRA are grounded in the views of different stakeholders especially as far the use of broad consent for genomics research is concerned (Jao et al., 2015). This suggests that more research is needed on the views of different stakeholders on the use of broad consent in genomics and bio-banking research in Zambia.

Our study is limited is two ways. Firstly, due to the time lag between the time the RHDGen study was implemented and the time we conducted our interviews, the participants were only telling us what they remembered about the study and there may have been some recall bias. We considered this in our study by focusing the interviews more on hypothetical questions relating to sample and data sharing that were not specifically linked to the RHDGen study. However, despite the time lag, almost all interviewees remembered enrolling in a research study exploring heart disease; that many described that the study related to inheritance or genetics of this condition; and that many recalled that samples were sent to South Africa. Secondly, most interviews were not conducted in English but in Bemba and Nyanja. This required, first, the translation of study materials (consent forms, topic guides) into these languages, and then the translation of recordings and transcripts into English. It also required the researchers conducting interviews to consider how to best describe pertinent terms and concepts in these languages. Several authors have described how difficult it is to effectively communicate about genomics research in African languages that lack terms for scientific concepts such as “genes”, “DNA”, “data sharing”, “bio-banking” and so forth (see for instance Tindana & De Vries, 2016; Tindana et al., 2012). We sought to be mindful of this challenge by not over-interpreting participants’ views, and by exploring the initial recording if we had questions about the accuracy of the translation.

Overall, our study contributes to the existing literature on broad consent in the context of genomics and bio-banking research. In particular, our study adds to the consensus that broad consent could be permissible and possible in African research settings, if explained well to participants, and if used in conjunction with restrictions on future use. It also adds to the debate on how broad consent is complicated in contexts where there are reciprocal interests by researchers and participants especially when participants are desperate for solutions to their medical problems. Lastly, it contributes to the debates on the appropriate regulations and legal framework for genomics and bio-banking research in Africa.
